# An Accessible Platform to Quantify Oxygen Diffusion in Cell‐Laden Hydrogels and Its Application to Alginate‐Immobilized Pancreatic Beta Cells

**DOI:** 10.1002/bit.70095

**Published:** 2025-11-11

**Authors:** Hamid Ebrahimi Orimi, Kurtis S. Champion, Laurier Gauvin, Jonathan A. Brassard, Berit L. Strand, Richard L. Leask, Corinne A. Hoesli

**Affiliations:** ^1^ Department of Chemical Engineering McGill University Montreal Quebec Canada; ^2^ Department of Biotechnology and Food Science, Norwegian Biopolymer Laboratory (NOBIPOL) Norwegian University of Science and Technology Trondheim Norway

**Keywords:** alginate, beta cells, cell immobilization, diabetes, hydrogel, oxygen diffusion

## Abstract

Hydrogels are commonly used to immobilize mammalian cells, offering mechanical support in 3D cultures and acting as barriers for immunoprotection in transplantation, such as islet encapsulation for diabetes therapy. Cell immobilization restricts bulk fluid motion, resulting in diffusion‐limited molecular transport and nutrient concentration gradients, particularly for oxygen consumed by immobilized cells. Oxygen mass transport models are essential for designing immobilization strategies but often rely on assumed diffusion coefficients due to a lack of experimental data. We propose a cost‐effective, accessible system for experimentally measuring oxygen diffusion coefficients in cell‐laden hydrogels, tested on alginate‐immobilized pancreatic beta cells (MIN6). Compared to water, oxygen diffusivity was significantly lower in alginate gels and inversely correlated with the dynamic loss modulus. Diffusivity also decreased with increasing alginate concentration from 2% to 5%. Cell viability depended heavily on gel concentration and cell density, as predicted by Thiele modulus and effectiveness factor values calculated from the measured diffusion coefficients. This platform, combining a simple experimental setup with dimensionless numbers, offers a practical way to predict maximal diffusion distances in cell immobilization strategies. The proposed approach can support rational design of cell encapsulation, immobilized cell culture, and tissue engineering strategies.

## Graphical abstract

A simple setup to measure oxygen diffusion coefficients was developed using equipment accessible to most wet laboratories. Gels that appeared softer and had lower loss moduli correlated with higher oxygen diffusion coefficients. Dimensionless number analyses using the Thiele Modulus and effectiveness factors were predictive of viability profiles within gels.

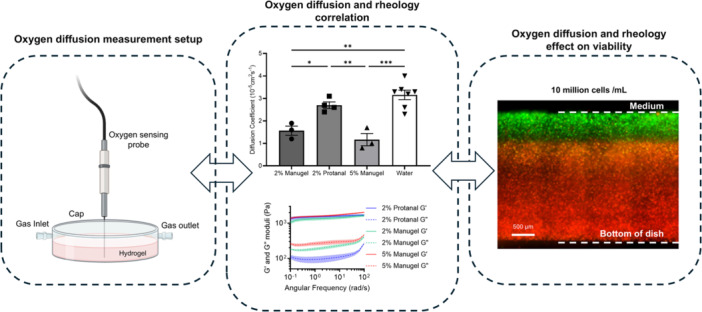

## 1 Introduction

Hydrogel‐based cell immobilization is used to provide mechanical support or stimuli to cells, to create organized tissue structures, or to create a barrier against harmful external conditions. Hydrogels such as alginate have been used to scale up the culture of mammalian cells (Hoesli et al. 2009; Smidsrod and Skjakbrk 1990; Thomas M.S. Chang 1964), in 3D bioprinting applications (Eswaramoorthy et al. 2021; Jia et al. 2014; Narayanan et al. 2016), as well as to retain or protect grafted cells in transplantation applications (Alagpulinsa et al. 2019; Swioklo et al. 2017; Trouche et al. 2010; Yu et al. 2010; Ziv et al. 2014). In their pioneering work, Lim and Sun demonstrated that pancreatic islets encapsulated in semipermeable alginate‐polylysine microcapsules could survive and restore normoglycemia in diabetic animal models, laying the foundation for the concept of a bioartificial pancreas (Lim and Sun 1980). The key principle behind this strategy is immunoprotection, where a semipermeable barrier allows diffusion of oxygen, nutrients, and insulin while preventing immune cell and potentially also antibody access to the graft (Vaithilingam et al. 2017). More recently, rapid progress in differentiating insulin‐producing β‐cells from human pluripotent stem cells (hPSCs) has provided potentially unlimited sources of therapeutic cells for transplantation (Pagliuca et al. 2014; Rezania et al. 2012). In this context, encapsulation is being revisited as a promising strategy not only to immunoprotect transplanted β‐cells but also to immobilize undifferentiated or off‐target cells, thereby reducing risks of tumorigenesis and enabling device retrieval in case of adverse events (Keymeulen et al. 2024). In all of these applications, the hydrogel can also create a barrier for bulk fluid motion, which can limit molecular transport of key nutrients, growth factors and functional proteins such as insulin, as well as waste products (Farina et al. 2019; Neumann et al. 2023; Shah 2013).

A common challenge in immobilized mammalian cell culture or transplantation is ensuring adequate oxygenation because of its low solubility in water and high rate of consumption, particularly when reaching tissue‐like cell densities. Oxygen gradients established by the balance of diffusion through the immobilization material and consumption by cells distributed within can result in cell dysfunction, hypoxia and death – reducing therapeutic potential.

Adequate oxygenation of encapsulated pancreatic islets has been a long‐standing engineering challenge (Bowers et al. 2019; Colton 2014; S. A. et al. 2022; Komatsu et al. 2018). In the native pancreas, islets exhibit an intricately structured angioarchitecture, essential for ensuring efficient oxygenation of all cells due to their pronounced aerobic metabolism (Bowers et al. 2019; Linn et al. 2006). After isolation from a donor, this native vasculature is disrupted. The cells at the core of avascular islet clusters rely on the external diffusion of oxygen making them much more vulnerable to hypoxia. This issue is exacerbated when encapsulating the cells in a hydrogel due to the addition of another external oxygen diffusion boundary (Bowers et al. 2019). Macroencapsulation, which involves delivering hundreds of thousands of islets (millions of cells) in a single device, can lead to substantial oxygen gradients because of local competition for oxygen (Moeun et al. 2025). These oxygen transport limitations are relevant not only to islet transplantation devices but also to any cell immobilization system where bulk fluid movement is restricted.

Several analytical and numerical models have been developed to predict oxygen gradients in islet and other cell encapsulation systems (Efstathios S. Avgoustiniatos and Colton 1997; Buchwald 2011; Cao et al. 2020; Johnson et al. 2009; Liang et al. 2021). Some of the critical parameters for these types of models include: the cell fraction, defined as the volumetric fraction of encapsulated cells relative to the total volume of the hydrogel or construct, the oxygen consumption rate (OCR) of the modelled cell(s), or the oxygen diffusion coefficient of the surrounding medium (ex: cell media, hydrogels, etc.). However, these modelling parameters are often assumed rather than experimentally determined, which can lead to inaccuracies and limit the implementation and reliability of such models (Al‐Ani et al. 2018). Quantifying parameters, such as the oxygen diffusion coefficient, is nontrivial but necessary to avoid substantial deviations expected and measured outcomes, especially in situations where oxygen diffusion dominates over oxygen consumption terms.

Oxygen diffusion coefficients can be determined experimentally by implementing probes or sensors placed either outside or within hydrogels. The simplest methods to determine gas diffusion coefficients through hydrogels are experimental setups in which gas flux through membranes (Higuchi 1984) or into gel particles (Mehmetoglu et al. 1996; Papas et al. 2007) is measured using sensors placed outside the gel. These methods typically rely on cell‐free, idealized model systems, such as thin gel layers (Adeoye and de Alba 2025). Oxygen probes can also be introduced directly into cell‐laden hydrogels, for example using needle‐type microsensors to enable minimally invasive, real‐time monitoring of oxygen gradients in thick constructs (Eggert et al. 2021). Local oxygen concentrations and oxygen diffusion coefficients can also be determined using molecular sensors that are oxygen‐sensitive and transmit signals either paramagnetically (Cristea et al. 2020; Kotecha et al. 2023) or optically (Bhunia et al. 2016; Figueiredo et al. 2018; Kotecha et al. 2023; Rharbi et al. 1999). These molecular sensors can either be directly embedded in hydrogels or first immobilized within nanoparticles or microparticles. Nanoparticle or microparticle immobilization can reduce probe leaching, enhance chemical and photostability, and improve signal intensity within hydrogels, leading to more reliable and long‐term oxygen sensing (Giuntini et al. 2014; Koduri et al. 2018; Lesher‐Pérez et al. 2017; Wang et al. 2013). Broadly, publications describing novel cell immobilization techniques often either do not model oxygen concentration gradients, or apply previously reported oxygen diffusion coefficients in transport models (Mavris and Hansen 2021)– likely because of the high cost, limited availability of suitable equipment, or need for custom probes required for many current direct measurement methods.

Here, we describe a simple and accessible setup comprised of an oxygen probe, sealed chamber, and gas supply to determine oxygen diffusion coefficients in liquids and hydrogels. The setup can be reproduced for approximately $2,000–$4,000 CAD (on Sept 22, 2025), depending on existing laboratory resources. The main cost comes from the oxygen probe and readout unit (e.g., OX500PT; PyroScience, approx. 400 CAD), whereas the other components—including the 3D‐printed cap, Petri dish, sealing film, fittings, and flowmeters—are relatively inexpensive. In contrast, commercial oxygen measurement systems, such as Clark‐type electrode chambers, typically cost around $5,400 CAD for single‐sample measurements, and advanced 3D oxygen imaging platforms based on fluorescence lifetime imaging microscopy (FLIM) can exceed $96,000 CAD. Our approach therefore provides a cost‐effective and accessible alternative that enables wider integration of oxygen diffusion estimates in biomedical research and engineering design. We demonstrate the application of this system to identify oxygen requirements of alginate‐immobilized pancreatic beta cells.

## 2 Materials and Methods

### 2.1 System to Measure Oxygen Diffusion Coefficient

**Figure 1:**
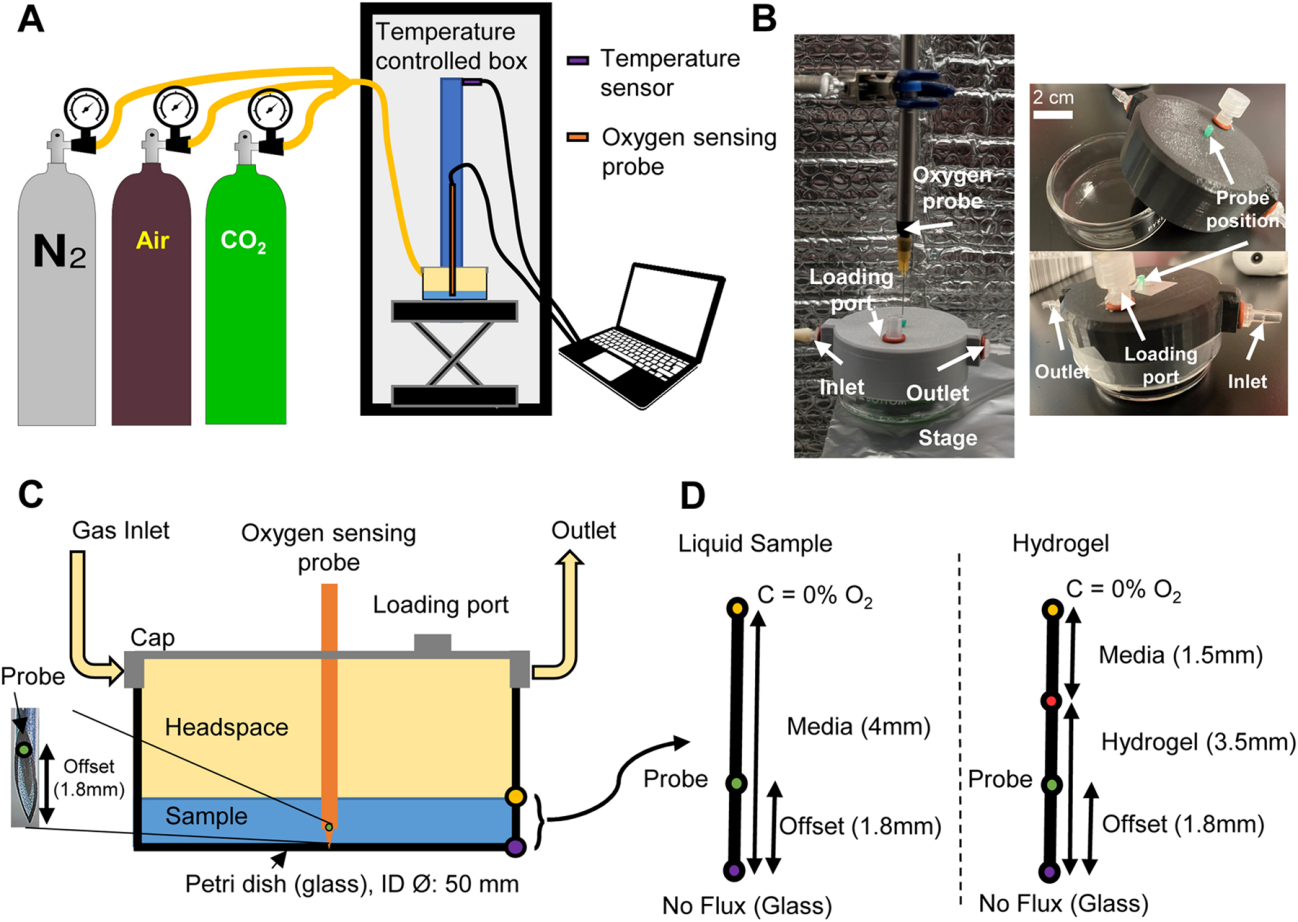
System setup and modelling approach. (A) Exterior system setup. Three blended gases (CO_2_, N_2_, Air) are fed into the Petri dish and oxygen is monitored. The setup is fully enclosed in a temperature‐controlled environment. (B) Photographs of the setup within the temperature‐controlled chamber, as the Petri dish system when open and sealed closed with sealing film. (C) Cross‐sectional schematic representation of the Petri dish setup with (D) relevant measurements and boundary conditions for modelling. The left‐hand panel in D shows distances for liquid samples (e.g. water) whereas the right‐hand panel shows distances for a hydrogel overlaid with liquid (e.g. cell‐laden alginate overlaid with culture medium).

The setup to measure oxygen diffusion is shown in Figure 1A. A 3D printed cap (see Supplementary Figures S1 and S2, Section 1) was attached to a glass Petri dish (316060; Pyrex) and sealed using sealing film (HS234526A, Sigma‐Aldrich) to control the oxygen tension at the surface while limiting oxygen diffusion at the bottom of the dish. This configuration was designed to force diffusion along a single vertical axis, enabling us to position the oxygen probe further away from the gas source while maintaining a consistent diffusion path. This design reduces potential deviations from unidirectional diffusion assumptions caused by inadvertent gas exposure or lateral diffusion and enhances the reproducibility of oxygen profile measurements. The positions for the loading port, inlet and outlet hoses were threaded and prepared by installing hose fittings (5117K83, McMaster‐Carr), which were sealed with O‐rings (5233T368, McMaster‐Carr). An oxygen sensing needle probe (OX500PT; Pyroscience) is fixed in place at the center of the system using a universal stand and a positioning mechanism (using a precision linear stage with 0.2 mm increment precision) allows for the local oxygen level to be measured at various depths (Figure 1B). To improve the measurement accuracy, the Petri dish setup is placed in a temperature‐controlled environment (Figure 1A and C). The gas entering the Petri dish is a mixture of air, N_2_ and CO_2_. CO_2_ was provided through volumetric control of a flowmeter (Cole‐Parmer; 03227‐04), while larger capacity flowmeters (Cole‐Parmer; 03227‐12) were used to regulate the amounts of both N_2_ and air.

The oxygen probe was first calibrated using a 2‐point calibration in the Pyroscience O2Logger software, the two points are: (i) 0% O_2_ using water with an oxygen scavenger (OXCAL, Pyroscience) and (ii) air‐saturated water at a set temperature in the software corresponding to the temperature measured with an alcohol thermometer. The temperature‐controlled box was set to 37°C, and all liquid or hydrogel samples were equilibrated in a cell culture incubator before testing (37°C; 5% CO₂). Inside the box, a mercury thermometer was used and verified to be within 0.5°C of the target before each experiment to verify the internal temperature. After placement into the measurement chamber, samples were maintained under the same controlled culture conditions (37°C; 5% CO₂) before nitrogen purging. Nitrogen flow was controlled using a manual flowmeter with a calibrated scale and control knob, and the flow rate of 300 mL/min was set directly based on the flowmeter's scale. On the gas line, before entry into the Petri dish, inline oxygen and CO₂ sensors (LuminOx flow‐through from SST DwyerOmega, factory‐calibrated and connected to manufacturer‐provided software) were used to verify the readings from the flowmeters. The gas line was purged at 300 mL/min for 10 min to eliminate dead volume effects, after which the flowrate was reduced to 130 mL/min. The OX500PT probe needle was then gently placed into the Petri dish sensing port.

A syringe with a 15‐gauge blunt‐edge needle was used to inject samples into the Petri dish using the loading port (Figure 1C). Initial gas bubbles were removed by flicking the probe. Oxygen measurements were continuously logged in the software with no data smoothing. After waiting 5–10 min for system stabilization (vibration, temperature, etc.), we attached the N_2_ purged gas line (130 mL/min) to the inlet (Figure 1C) and captured the gradual decrease in oxygen over time. An experimental check using water was performed for hydrogel experiments to confirm diffusivity accuracy. Figure 1D illustrates the dimensions and positioning of the probe within liquid or hydrogel samples.

### 2.2 Further Assessment of Oxygen Diffusion Experimental Setup Using PDMS‐CaO_2_ Slabs

To assess the effectiveness of the oxygen diffusion setup proposed in this study, we quantified the oxygen release rate in slabs composed of polydimethylsiloxane (PDMS) and calcium peroxide (CaO_2_). To prepare the composite, we mixed PDMS base, curing agents (10:1 v/v ratio), and 10% w/v CaO_2_, and then subjected the mixture to vacuum for 20 min to remove air bubbles. The resulting mixture was poured into a glass petri dish and cured in an oven at 50°C for 12 h. Then the slab was removed from the mold and placed at the bottom of the device before placing the cap and sealing.

### 2.3 Computational Modelling and Governing Equations

All modelling was performed in COMSOL Multiphysics 5.6. The modelling was performed in two steps: (1) the boundary condition verification which makes use of the 2D time independent laminar flow module, and (2) the diffusion coefficient numerical model which makes use of the 1D time dependent transport of dilute species module.

### 2.4 Boundary Condition Verification Using Computational Fluid Dynamics

The 2D laminar flow module was used to model the gas flow profile in the headspace between the hydrogel/fluid and the cover of the glass Petri dish. In turn, this model can estimate the water surface velocity and provide an estimate for the Péclet number to determine if the system is diffusion driven. A 2D geometry was used for improved mesh resolution and corresponds to the center plane of the Petri dish where the oxygen probe is located. The simulation assumes incompressible fluid, steady fully developed laminar flow at the Petri dish inlet with the flow rate as set via the gas flow meter, no slip condition at the Petri dish rigid wall, Newtonian fluid, and incompressible fluid (air and liquid region). The boundary layer at the surface of the liquid was taken as an air‐liquid interface. The boundary conditions are: (1) normalized laminar flow at the inlet (flowrate per known inlet length scale). (2) zero pressure at outlet (results in gauge pressure profile), and the model is at steady state (time independent). Note that the length scale used to normalize the flowrate was determined by the inner diameter in the inlet tubing, which is 4 mm. An “extremely fine” mesh was used for all calculations (45830 elements; size of 5 × 10^−4^ to 0.168). The governing equations for solving the velocity and pressure profile were the 2D cartesian time independent Navier‐Stokes equation, and the conservation of mass (continuity equation). The shear stress profile was calculated from the product of the shear rate and viscosity (both calculated by COMSOL natively).

The Péclet number (Pe) is the ratio of advective mass transport to diffusive mass transport. It was calculated using the perpendicular surface velocity (u) found from the model at the air‐liquid interface boundary layer, the characteristic length (L) which was taken as the height of liquid sample, and the diffusion coefficient (D) (Equation 1).

(1) $$\mathrm{Pe}=\frac{\mathrm{advective transport}}{\mathrm{diffusive transport}}=\frac{\mathrm{uL}}{D}$$

Pe < 1 in general suggests that the system is diffusion driven and that recorded data is predominantly a result of diffusion.

### 2.5 1D Time Dependent Mass Transfer Model

The 1D transport of dilute species module in COMSOL 5.6 was used to model the oxygen depletion across the center axis of the Petri dish where the oxygen probe is located. The boundary conditions for this line are presented in Figure 1D. An extremely fine mesh (45830 elements; size of 5 × 10^−4^ to 0.168) was used to solve all numerical models, and all reaction terms were ignored since these experiments were acellular. The governing equation for this modelling is Fick's law (Equation 2):

(2) $$D\frac{\partial^{2}c_{O_{2}}}{\partial z^{2}}=\frac{\partial c_{O_{2}}}{\partial t}$$

Where z is the length along the center axis where the probe is located.

**Figure 2:**
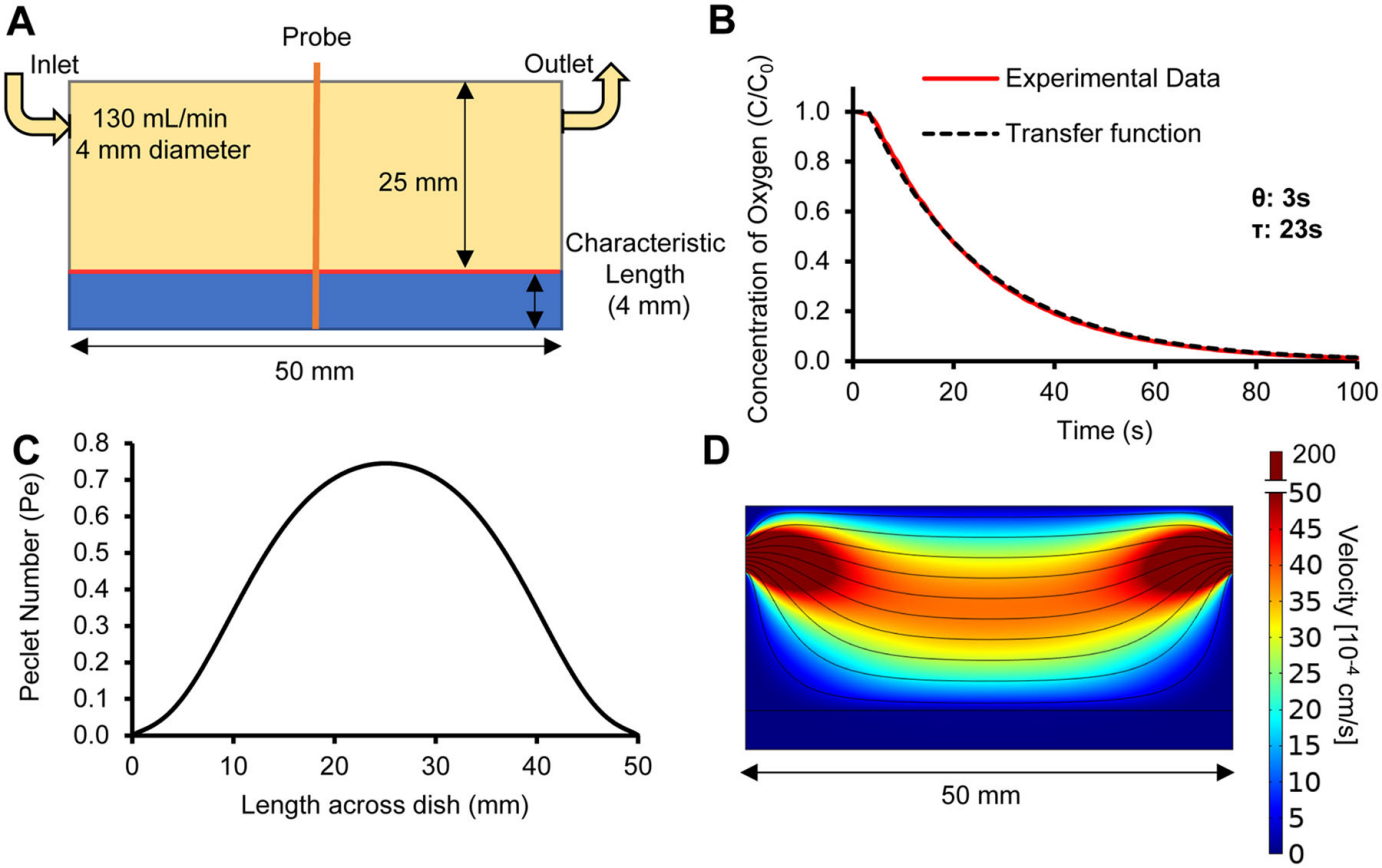
Mass transfer modelling boundary condition justification and assessing advective effects of headspace flow profile. (A) Geometric and gas flow conditions applied in a finite element model of gas and liquid velocities within the system The red line indicates the gas‐liquid interface. (B) Experimental oxygen concentration response measured at the air/liquid boundary (4 mm distance from the dish bottom) after applying a step change from 21% to 0% at the gas inlet, normalized to the initial concentration (C_0_). The response was modeled through a first‐order transfer function with a time delay (θ) of 3 s and a time constant (τ) of 23 s. (C) The Péclet number (Pe) along the gas‐liquid interface based on finite element modeling. Pe values were calculated using the perpendicular velocity component (u), the characteristic length (L = 4 mm), Pe = uL/D_water_ where D_water_ = ~3.1 × 10^−5 ^cm^2^/s. Average Pe is 0.48 < 1, max Pe is 0.75 < 1. (D) Velocity magnitude profile (colour map) and streamlines (black) at the cross‐section based on finite element modeling.

For a liquid, the top of the line in contact with the gas‐controlled boundary condition is assumed to have an oxygen concentration of 0 (C = 0 mol/m^3^) or to follow the transfer function experimentally determined in Figure 2B at the appropriate liquid height. The transfer function accounts for the time delay between a step change in oxygen at the inlet and the concentration at the probe location. The opposite end in contact with glass is assumed to have a no flux boundary condition. This assumption is valid since glass has an oxygen diffusion coefficient that is orders of magnitude lower than water and hydrogels. For hydrogel modelling, an additional interface exists between the top up media and hydrogel as shown in Figure 1D. Media is assumed to have the same diffusivity as water at 37°C. The probe offset location is determined experimentally and corresponds to where the model is compared to experimental data. The offset distance from the tip of the needle to the beginning of the probe (sheathed within the needle) was measured using a caliper and microscope.

### 2.6 Thiele Modulus and Effectiveness Factor

The Thiele modulus (φ) describes the relationship between the reaction rate and the diffusion rate in mass transfer for porous materials like hydrogels. The oxygen consumption in islets was assumed as a 0th order reaction where the cells fixed oxygen consumption rate.0th order kinetics can be assumed if the ratio between the Michaelis–Menten coefficient (K_m_) and surface concentration (C_s_) approaches 0 ($C_{s}\gg K_{m}$) (AL‐Muftah and Abu‐Reesh 2005). This assumption applies to all cells involved in this study, as the oxygen levels never fall below 0.03 mL/m³ (~25 mmHg), which is higher than the $K_{m}$ of islets (as detailed in the supporting materials, Figure S3, Section 2). Islets have a Michaelis–Menten coefficient of ~0.44 mmHg for oxygen consumption (Avgoustiniatos and Colton 1997; Buchwald 2011) which is much less than the surface oxygen tension of at least 40 mmHg (Olsson and Carlsson 2011) observed for immobilized islet systems when transplanted into an environment where the device surface is exposed to oxygen tensions at the lower end of venous values.

In this study, oxygen transport within the hydrogel is modeled under steady‐state conditions, assuming one‐dimensional diffusion through a homogeneous, isotropic, and non‐swelling material. At the hydrogel surface (x = 0), the oxygen concentration is fixed and equal to the external concentration. At a specific depth within the hydrogel (x = x_crit_), the boundary condition is either a known critical oxygen concentration or a zero‐flux condition, depending on the desired formulation. These assumptions allow estimation of oxygen penetration depth and assessment of whether oxygen supply remains above the critical threshold for cell viability.

The Thiele modulus for a zero‐order system was derived based on a constant reaction rate (k_v_) equal to the cell specific oxygen consumption rate (OCR) multiplied by the cell concentration (X), and surface oxygen concentration (C_s_) and is presented as the following (Equation 3):

(3) $$\varphi =\frac{\mathrm{reaction rate}}{\mathrm{diffusion rate}}=L\sqrt{\frac{k_{v}}{D_{\mathrm{eff}}C_{s}}}=L\sqrt{\frac{X\times \mathrm{OCR}}{D_{\mathrm{eff}}C_{s}}}$$

Where L is the thickness.

The effectiveness factor was calculated for a slab geometry with a zero‐order reaction (Equation 4).

(4) $$\eta =\left\{\begin{array}{l} 1;\;\varphi \leq \varphi_{\mathrm{crit}}\\ \frac{1}{\varphi }\sqrt{2\left(1-\frac{C_{\mathrm{crit}}}{C_{s}}\right)};\;\varphi >\varphi_{\mathrm{crit}} \end{array}\right.$$

### 2.7 Adherent Cell Culture

Mouse insulinoma 6 (MIN6) cells (generously gifted from Dr. Jun‐ichi Miyazaki‐ Osaka University (MIYAZAKI et al. 1990)) in complete medium were seeded in tissue culture treated polystyrene flasks until 80%–90% confluency. The complete medium consisted of Dulbecco's Modified Eagle medium (DMEM; Gibco #10313‐021, Thermo Fisher Scientific) supplemented with 10% fetal bovine serum (FBS; HyClone #SH3039602, Fisher Scientific), 1% l‐glutamine (Gibco #25030‐081, Thermo Fisher Scientific), 1% penicillin/streptomycin (Gibco #15140‐122, Thermo Fisher Scientific), and 0.1% 2‐mercaptoethanol (Fisher Chemical #O3446I, Fisher Scientific). Changes were performed every 48–72 h. An incubator at 37°C and 5% CO_2_ was used to culture the cells. Passaging was performed using 1X TrypLE Express Enzyme (Gibco #12605‐028, Thermo Fisher Scientific). Experiments were conducted at passage number between 28 and 40.

### 2.8 Production of Alginate Slabs

Alginate slabs were produced using internal gelation. The two types of alginate used in this study were Manugel GHB (IFF Nutrition & Biosciences (formerly FMC Biopolymer) through DuPont) and Protanal 10/60 LF (IFF Nutrition & Biosciences (formerly FMC Biopolymer) through DuPont). A stock solution of either 2.5% w/v Manugel GHB, 2.5% w/v Protanal, or 6.25% w/v Manugel GHB was prepared in pH 7.4 HEPES‐buffered saline (170 mM NaCl; 10 mM HEPES). The alginate stock solution was autoclaved at 121°C for 30 min; 100 mL of the stock solution was autoclaved each time. A second stock solution of 0.5 M calcium carbonate (CaCO_3_; Avantor #1301‐01, VWR) was also prepared in pH 7.4 HEPES‐buffered saline and sonicated for 15 min to disperse calcium carbonate particles. Thereafter, this solution was autoclaved at 121°C for 30 min. Glucono‐δ‐lactone (GDL; Sigma‐Aldrich #G4750, MilliporeSigma) was dissolved in HEPES‐buffered saline and sterilized with a 0.2‐µm nylon syringe filter (Fisherbrand™ #09‐719 C, Fisher Scientific) immediately before use.

To prepare a slab with a height of at least 4 mm with a 50 mm diameter, at least 5.6 mL of alginate solution is necessary. For all experiments, 10 mL of alginate hydrogel mixture was made. To do so, 0.6 mL of the 0.5 M CaCO_3_ solution was added into 8 mL of 2.5% alginate or 6.25% alginate and mixed using a sterile spatula in a 10 mL syringe with the plunger removed and the bottom capped. A 1 mL solution of cell culture media containing trypsinised MIN6 cells that will result in the desired final cell concentration was then added to the syringe and mixed using the spatula. Finally, 0.6 mL of the GDL solution was added to the syringe. The resulting solution is ~2% or ~5% w/v alginate stock, 30 mM CaCO_3_, and 60 mM GDL. The syringe plunger was then reintroduced, while preventing any alginate solution from leaking out. The solution, 7.9 mL, was injected and evenly spread into glass Petri dish (Ø 50 mm). Note that 7.9 mL corresponds to a 50 mm cylinder with a 4 mm height (characteristic length used for Pe and modelling). Hydrogel samples include a small amount of top up media to prevent the gel from drying out.

The slabs were left to gel between 30 and 40 min (until visually solid) and topped up with 4 mL of cell culture media to prevent the slab from drying out. The Petri dish was then sealed with a piece of parafilm that was previously soaked in ethanol. The slabs were placed in an incubator at 37°C and 5% CO_2_ for 16–24 h.

### 2.9 Live/Dead Staining & Image Acquisition

After the slabs underwent 16–24 h of incubation, a very thin vertical slice of the center of the slab was cut using a razor blade and stained using a live/dead solution with final concentrations consisting of 18.9 µg/mL propidium iodide (Fisher Scientific; P1304MP) and 1.1 µg/mL Calcein AM (Fisher Scientific; C1430) both in pH 7.4 HEPES‐buffered saline at room temperature. Images were acquired using an IX81 Olympus Microscope at 10X magnification on the full chip setting using a FITC filter cube (Ex: 482/35 | Em: 536/40) and a Texas Red filter cube (Ex: 525/40 | Em: 585/40). The exposure time used for all imaging was 100 ms.

### 2.10 Image Analysis for Live/Dead Staining

Images were stitched in a grid‐wise format using a stage file generated in MATLAB (Liu 2023), a step size of 850 μm in the X and Y direction. Live/dead fluorescence analysis and live MIN6 cells depth analysis was performed in FIJI (ImageJ). Acquired images were cropped from 2048 × 2048 pixels to 1550 × 1550 pixels and stitched column‐by‐column, up‐and‐right, with an overlap of 18% using the built‐in stitching function (Preibisch et al. 2009). FITC and Texas Red images were merged where FITC was chosen to be green and Texas Red which was red. Live MIN6 cells depth analysis was performed by setting a calibrated scale of 1300 pixels:850 μm and by visually measuring the live height (the green portion) and total slab height at the top, middle, and bottom of the slab using the line tool in ImageJ.

### 2.11 Alginate Characterization

Alginate composition was characterized by ^1^H‐NMR as described previously (Ertesvåg and Skjåk‐Bræk 1999; Grasdalen 1983; Grasdalen et al. 1979). Alginate solutions were prepared in HEPES‐buffered saline containing 170 mM NaCl and 10 mM HEPES, adjusted to pH 7.4. The alginate was dissolved in HEPES‐buffered saline to form a stock solution, which was then sterilized by autoclaving at 121°C for 30 min. To eliminate the influence of salts present in the buffer, the solution was dialyzed extensively against ultrapure water using pretreated regenerated cellulose dialysis tubing (Repligen Spectra/Por 7, 45 mm flat width, molecular weight cutoff [MWCO] 1 kDa, 132105, Spectrum Laboratories Inc.). Following dialysis, the alginate samples were lyophilized and stored at 4°C, then shipped at ambient temperature from Montreal (Canada) to Trondheim (Norway) for analysis. For analysis, the alginate was degraded by acid hydrolysis and lyophilized. 5–10 mg dry sample was dissolved in 600 μL D2O (99.9%) and added 5 μL 3‐(Trimethylsilyl) propionic 2,2,3,3‐d4 acid (TSP, Sigma Aldrich) as an internal standard. 1D spectra was recorded at 90°C on a 600 MHz spectrometer (Bruker BioSpin AG, Fällanden, Switzerland). The spectra were recorded using TopSpin 1.3 or 2.1 software and processed and analyzed with TopSpin 3.0 software (Bruker BioSpin). The anomeric protons in alginates have chemical shifts in the region 4.3–5.3 ppm, and fractions of monomers (F_G_, F_M_), dimers (F_GG_, F_MM_, F_GM, MG_), trimers (F_GGG_, F_MGM_, F_GGM, MGG_) and minimum G‐block length (N_**G>1**_) was calculated from the intensity profiles in this region as described previously (Ertesvåg and Skjåk‐Bræk 1999; Grasdalen 1983; Grasdalen et al. 1979).

The weight average molecular weight (M_W_), number average molecular weight (M_n_) and polydispersity index (M_w_/M_n_) of the alginate samples was analyzed by Size Exclusion Chromatography with Multi Angle Light Scattering (SEC‐MALS) (Vold et al. 2006). Samples and standards were dissolved in the mobile phase (0.05 M Na_2_SO_4_ with 0.01 M EDTA, pH 6.0) and filtered (0.8 μm). Astra software v. 6.1 (Wyatt, USA) was used to collect and process the data obtained from the light scattering and the differential refractometer, using a refractive index increment (at constant chemical potential), (dn/dc)_μ_ of 0.150 mL/g (Vold et al. 2006).

### 2.12 Rheology

A modular compact rheometer (MCR 302, Anton Paar) was used to measure the storage modulus (G’) and loss modulus (G”) of hydrogels composed of 5% (w/v) Manugel, 2% (w/v) Manugel, and 2% (w/v) Protanal. The hydrogels were fabricated into slabs approximately 25 mm in diameter and 2 mm in thickness using internal gelation approach. Hydrogel slabs were incubated in Dulbecco's Modified Eagle Medium (DMEM) at 37°C with 5% CO_2_ and humidity for 24 h.

To study the shear stress experienced by cells, the hydrogel slabs were compressed with a constant axial force (0.2 N) to prevent slipping and subjected to an oscillation strain of 0.5%, while varying the applied angular frequency between 0.1 and 100 rad/s. Furthermore, the hydrogel's elasticity was investigated under an amplitude sweep by keeping the strain rate constant at 10 s^‐1^ and varying the oscillation amplitude from 1% to 100%.

### 2.13 Statistical Analyses

All averages and standard deviations were computed using Microsoft Excel. The data was plotted in either Excel or in GraphPad Prism (9.1.2). All data underwent normality checks in GraphPad utilizing the Shapiro‐Wilk test. To evaluate differences between experimental groups, a one‐way ANOVA was performed, followed by the Tukey test to compare groups. Oxygen diffusion coefficients were determined by minimizing the mean absolute percent error (MAPE) of measured oxygen concentration profiles as a function of time to numerical predictions as determined by the 1D time dependent model.

## 3 Results

A straightforward way to measure the oxygen diffusion was developed in this study. Using an oxygen probe and conventional laboratory equipment, the oxygen diffusivity of various hydrogel formulations was measured.

### 3.1 Numerical and Experimental Validation of the Platform

The gas flow rate applied (Figure 2A) impacts oxygen profiles within the system. If the gas flow rate is too low, a significant delay between the oxygen concentration present at the air/liquid boundary and the concentration applied to the system may arise. If the gas flow rate is too high, it may disturb the gas/liquid interface leading to fluid motion and oxygen transport by advection. At the gas flow rate selected (130 mL/min), which corresponds to ~2.65 full gas exchanges/min, less than 2 min are needed for the gas concentration measured at the air/liquid interface to reach the concentration applied at the system inlet (Figure 2B). As compared to the 1~2 h experimental times, the time required for the boundary to reach the desired concentration is negligible. Based on finite element modeling, the Peclet number (Pe; ratio of advection to diffusion) remained below unity across the system, suggesting that oxygen transport is dominated by diffusion (Figure 2C). Visual inspection also indicated that there was no significant fluid motion upon applying gas flow. In these conditions, a uniform oxygen concentration profile was observed across the central area of the Petri dish, where the oxygen probe is positioned (confirmed by the simulation presented in Figure 2D).

**Figure 3:**
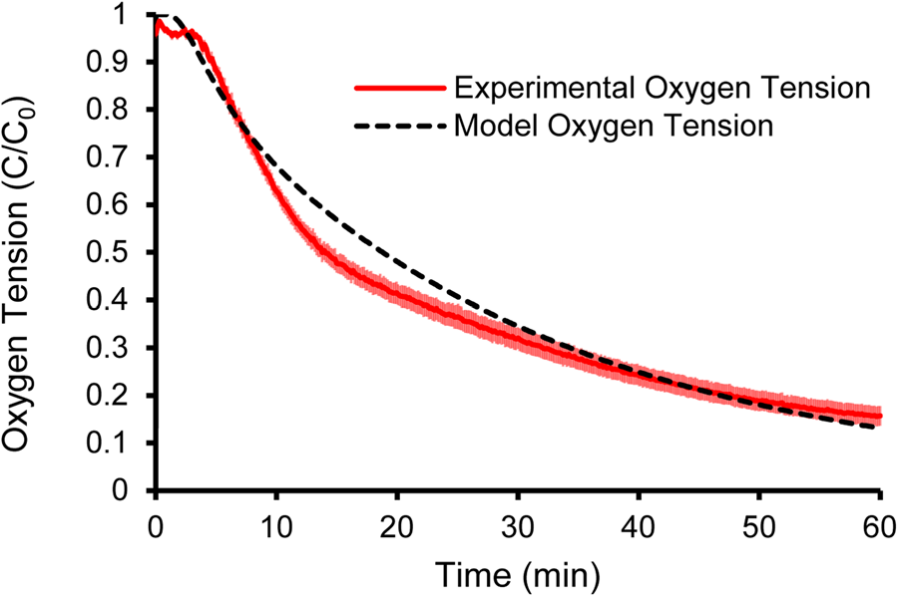
System validation using water at 37°C. Experimental nondimensional concentration data (red) of oxygen depletion in air‐saturated water (*n* = 7). Numerical model with diffusion coefficient of water at 37°C (D ~ = ~3.1 × 10^‐5^ cm^2^s^‐1^) Error bars: SEM, *N* = 7 experimental replicates.

To further validate the system, the diffusion coefficient of water at 37°C was estimated using the setup and was compared to literature values which range from 2.9 × 10^−5^ −3.3 × 10^−5^ cm^2^s^−1^ (Al‐Ani et al. 2018; Buchwald 2009; Noël and Mauroy 2019; Wilke and Chang 1955; Xing et al. 2014). To estimate the diffusion coefficient from experimental measurements, the oxygen concentration response measured by the probe (center, bottom of the Petri dish) was compared to the unidimensional time‐dependent mass transport model (Figure 3) to extract the diffusion coefficient providing the best fit. The response observed experimentally showed faster initial response than expected from finite element modeling, which may reflect some advection effects. The impact of advection would be reduced further for hydrogels where oxygen diffusion coefficients are lower, and diffusion will become more dominant. Water diffusion coefficient values were acquired before each alginate measurement, and results were reproducible. The value obtained was (3.3 ± 0.23) ×10^−5^ cm^2^s^−1^, which is within the reported range.

To assess the experimental system's capacity, a combination of PDMS and CaO_2_ was utilized as an oxygen producer. This method enabled the quantification of the amount of oxygen generated during oxygen purging in the setup. The oxygen release profile of the CaO_2_/PDMS beads is presented in the supporting materials, alongside the oxygen release profile of the PDMS beads (supporting materials, Figure S4, Section 3).

In addition to oxygen diffusion coefficient estimates, the system could be of interest to study oxygen release kinetics from oxygen‐generating materials (Coronel et al. 2019). PDMS microbeads, with or without CaO_2_ loading, were added to the water sample. As expected, slow hydrolysis of the CaO_2_ led to an increase in oxygen concentration during N_2_ purging of the system (supporting information Figure S4, Section 4).

### 3.2 Alginate Oxygen Diffusion Coefficient Measurement

**Figure 4:**
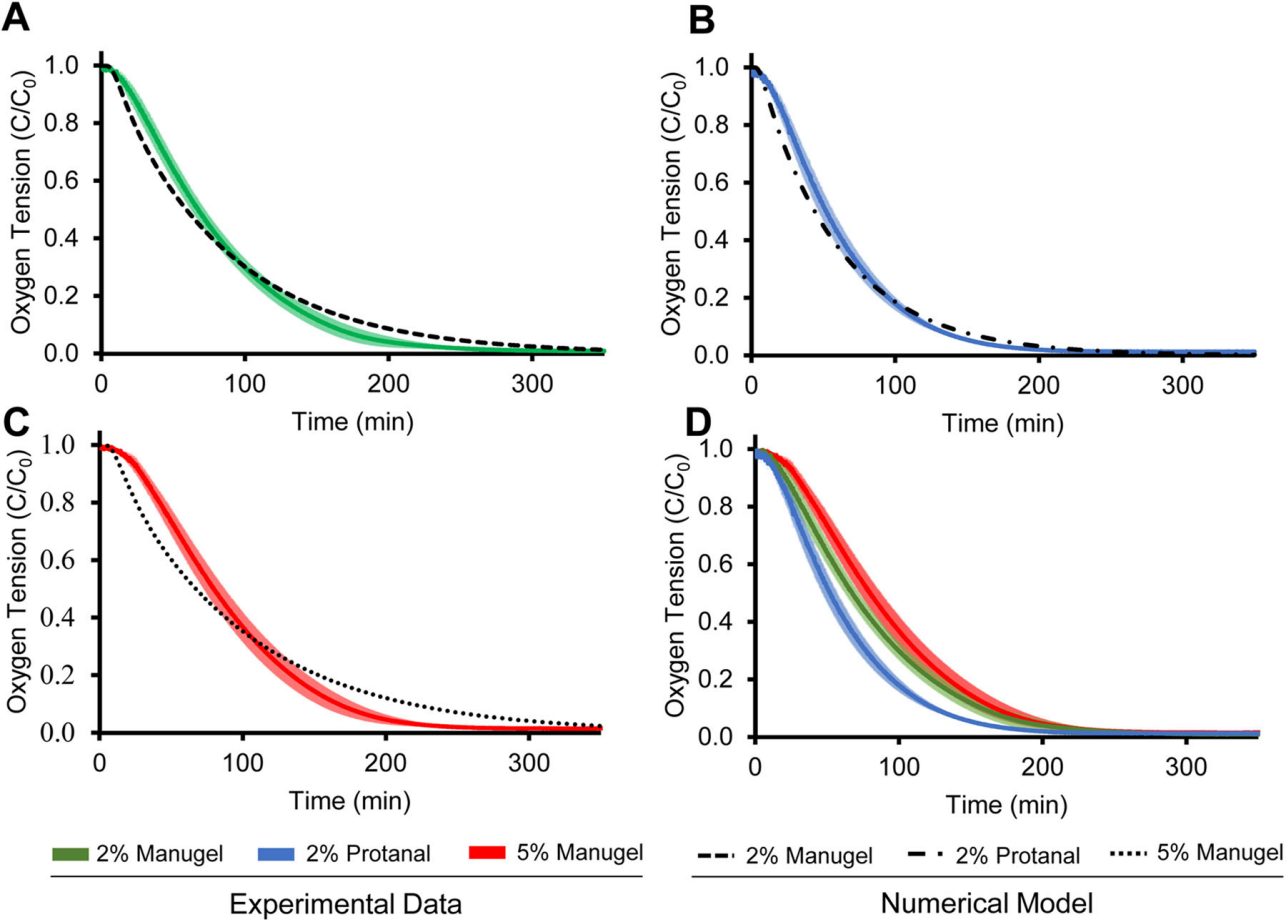
Diffusion coefficient of different alginate gels at 37°C. Oxygen depletion from 21% to 0%, normalized to initial concentrations for (A) 2% w/v Manugel, (B) 2% w/v Protanal alginate and (C) 5% Manugel. (D) Overlay of experimental data of all alginate formulations tested. Error bars: SEM, *N* = 3 to 4 experimental replicates. Dashed lines indicate the best fit to the data based on COMSOL simulation results.

**Figure 5:**
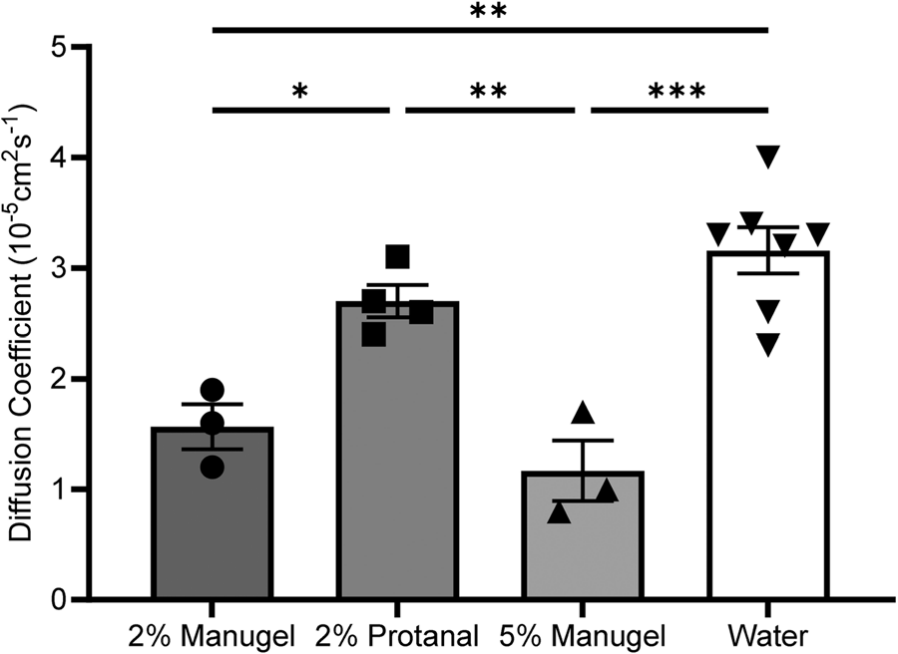
Comparative analysis of diffusion coefficient of various alginate compositions at 37°C. Diffusion coefficients were obtained by minimizing the mean average percent error between experimental data and the unidimensional time‐dependent mass transport model. Experimental replicates: *n* = 3 for 2% Manugel and 5% Manugel, *n* = 4 for 2% Protanal, and *n* = 7 for water. Significance was assigned as followed: *p ≤ 0.05, **p ≤ 0.01, ***p ≤ 0.001. Error bars: SEM, *N* = 3 to 4 experimental replicates.

The system was then used to measure the experimental oxygen diffusion coefficients of different formulations of alginate. As for water, the fitted and experimental oxygen concentration response after a step decrease in oxygen at the system inlet followed similar but not identical trends. Experimental responses were initially delayed as compared to model fit, particularly for the higher‐concentration alginate condition. This is characteristic of systems with Pe numbers above 1 (Arora and Potůček 2009; Ganaie et al. 2013), and could be due to radial diffusion effects neglected by the model that would be more prominent in gels with lower diffusion rates. The gel with the slowest response was the 5% Manugel formulation, followed by the 2% Manugel and the 2% Protanal gel, as also reflected by the fitted diffusion coefficient values (Figure 4 and Figure 5). There was no significant difference between water and 2% Protanal. Although the diffusion coefficient differences between 5% and 2% Manugel did not reach statistical significance, the faster trend observed in the raw data may indicate an effect of the polymer concentration – as would be expected for more tortuous diffusion paths created by polymer chains (Bellich et al. 2011; Najdahmadi et al. 2018). However, this effect was not as important as the difference between polymer sources, highlighting the limitation of using literature values when estimating alginate diffusion coefficients.

To investigate whether the differences in oxygen diffusion coefficients between Manugel and Protanal could be attributed to variations in guluronic acid (G) content, molecular weight, or the abundance of G‐blocks, we analyzed the chemical composition of the respective alginate gels. Surprisingly, both types of alginate displayed very similar overall compositions, suggesting that the observed differences in oxygen transport are unlikely to result from gross compositional differences (Table 1).

**Table 1:** Molecular Weight, M/G Ratio, and Chain Analysis of Alginate Variants Manugel GHB and Protanal 10/60 LF, in Powder and Autoclaved Forms (F represents the mole fraction).

Alginate	Molecular weight			M/G ratio	Mannuronic acid (M)/guluronic acid (G) Chain Analysis								
	M_w_ (kDa)	M_n_ (kDa)	M_w_/M_n_		F_G_	F_M_	F_GG_	F_GM, MG_	F_MM_	F_GGM, MGG_	F_MGM_	F_GGG_	N_G>1_
Manugel GHB (powder)	176	73	2.4	0.47	0.68	0.32	0.57	0.11	0.21	0.041	0.071	0.53	15
Autoclaved Manugel GHB (2% w/v)	90	43	2.1										
Protanal 10/60 LF (powder)	143	64	2.2	0.49	0.67	0.33	0.55	0.11	0.22	0.039	0.078	0.52	15
Autoclaved Protanal 10/60 LF (2% w/v)	82	41	2.0										

However, this similarity may, in part, be influenced by the dialysis process used during sample preparation. The use of regenerated cellulose dialysis tubing with a 1 kDa molecular weight cutoff could have removed low molecular weight fractions including impurities differentially between the two alginate types. Moreover, subtle structural features—such as the regional distribution of G‐ and M‐blocks or differences in chain conformation—may not be captured by bulk compositional analysis. CaCO_3_ dissolution and alginate internal gelation will be affected by pH and buffer capacity of the system. Subtle differences in alginate composition may therefore impact alginate chain interactions during and after gelation, thereby changing molecular diffusion through gels.

**Figure 6:**
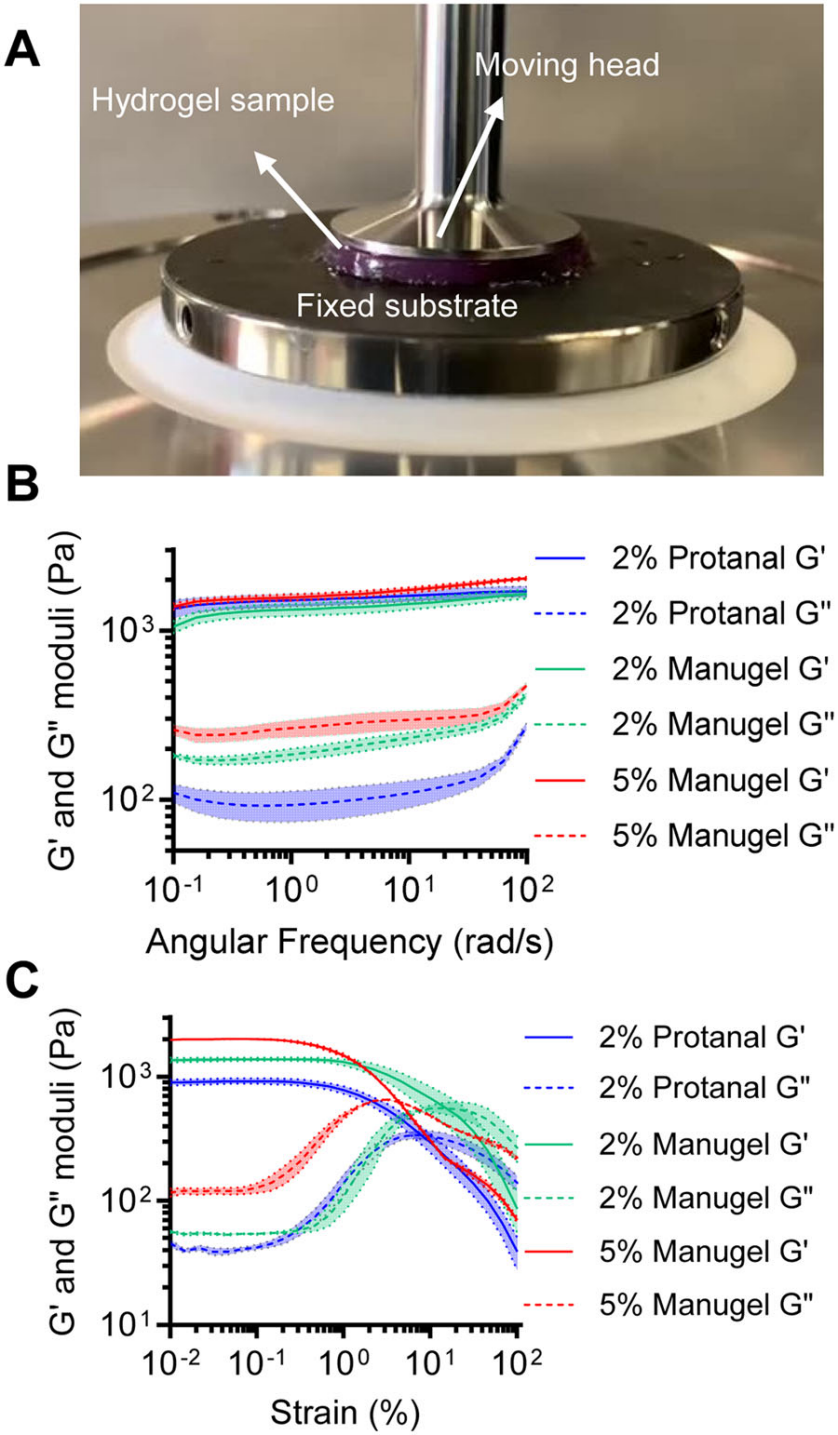
Frequency sweep experiment in rheology of hydrogels at room temperature. (A) The hydrogel sample placed under the rheology machine. (B) Experimental storage and loss modulus versus oscillation frequency (0.1 to 100 Hz). (C) Experimental storage and loss modulus versus strain (0.01% to 100%) for 5% w/v Manugel alginate (red zone), 2% w/v Manugel alginate (green zone) and 2% w/v Protanal alginate (blue zone). Error bars: SEM, *N* = 3 experimental replicates.

Qualitatively, we had observed that the Protanal gels, at the same concentration, appeared softer than the Manugel gels. We therefore hypothesized that rheological properties may correlate with oxygen diffusion coefficients. Our results showed that the loss modulus was lowest for 2% Protanal, followed by 2% Manugel and 5% Manugel, independent of whether amplitude or frequency sweep tests were performed (Figure 6B and C). This trend was consistent with the oxygen diffusion rates, with higher estimated oxygen diffusion coefficients observed for gels with lower loss moduli, and thereby lower hydrogel viscosities. The loss modulus indicates the viscosity of the hydrogel, with higher values indicating greater viscosity. Slight differences were found between the storage modulus values of the three hydrogels. To assess the significant differences in elasticity among the hydrogels that explain their varying softness, a rheological test was conducted using varying oscillation amplitudes. This test revealed differences in the amount of stored energy (Figure 6C) (the shear stress plot can be found in the supporting materials, Figure S5, Section 4).

### 3.3 MIN6 Cell Viability and Theoretical Cell Fractions Based on Thiele Modulus

**Figure 7:**
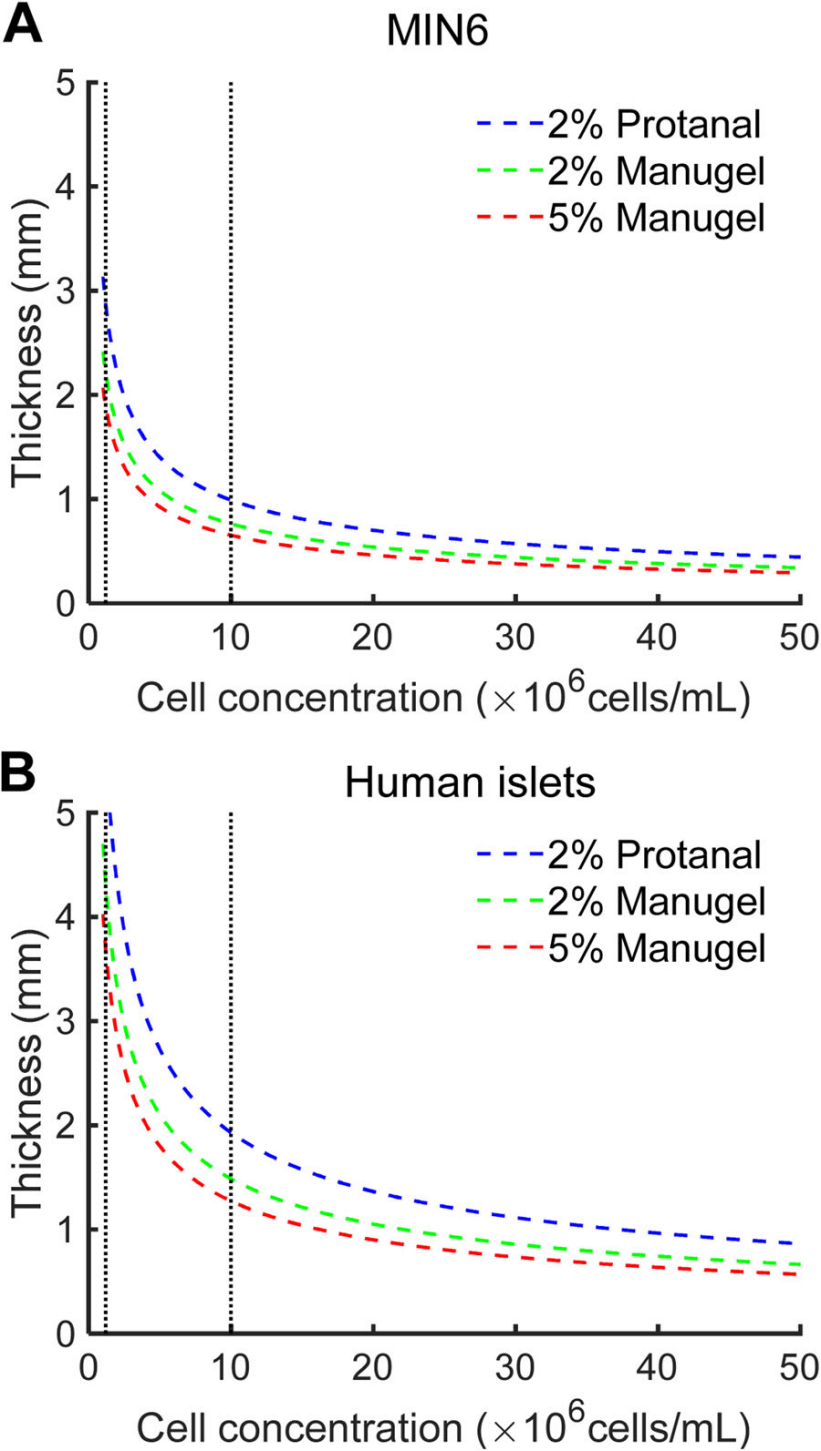
Theoretical hydrogel thickness versus cell concentration of (A) MIN6 cells and (B) human beta cell to achieve an effectiveness of 0.9 (η = 0.9).

Since the experiment was done in a simple 1D slab geometry, the effective length (L) is the height of the gel (3.5 mm as per Figure 1D). As shown in Figure 1C and Figure 1D, due to the height of the needle tip attached to the oxygen probe, oxygen measurements are taken at a position offset by 1.8 mm from the needle tip or the bottom of the Petri dish. The diffusion (D_eff_) was taken as a weighted average using the cell fraction between the diffusion coefficient of tissue (1.24 × 10^−5 ^cm^2 ^s^−1^ (Buchwald 2009; Johnson et al. 2009)) and the diffusion coefficient of the material as per Figure 5. The OCR used for MIN6 cells is 0.129 mol·m^−3^ s^−1^ (S. A. et al. 2022) and 0.034 mol·m^−3^s^−1^ (Buchwald 2011; S. A. et al. 2022; Johnson et al. 2009; Liang et al. 2021) for human islets, note that the MIN6 cells used are from the same cell stock used in (S. A. et al. 2022). The oxygen surface tension will not always be exactly 18.6% O_2_ since there is still a layer of top up media approximately 1.5 mm high that oxygen must diffuse through before reaching the top of the slab (as per Figure 1D). To determine the surface oxygen levels of the slab, a separate numerical model (as detailed in the supporting materials, Figure S6, section 5) was made to develop a relationship between the surface oxygen tension (C_s_) and the cell fraction (X) for a given OCR (MIN6 cells or islets) and alginate diffusion coefficient. Finally, the required hydrogel thickness was calculated for varying cell concentrations (X) to achieve an effectiveness factor of 0.9. This provides a conservative estimate of the maximum permissible cell density for a given encapsulation geometry and material. (Figure 7).

**Figure 8:**
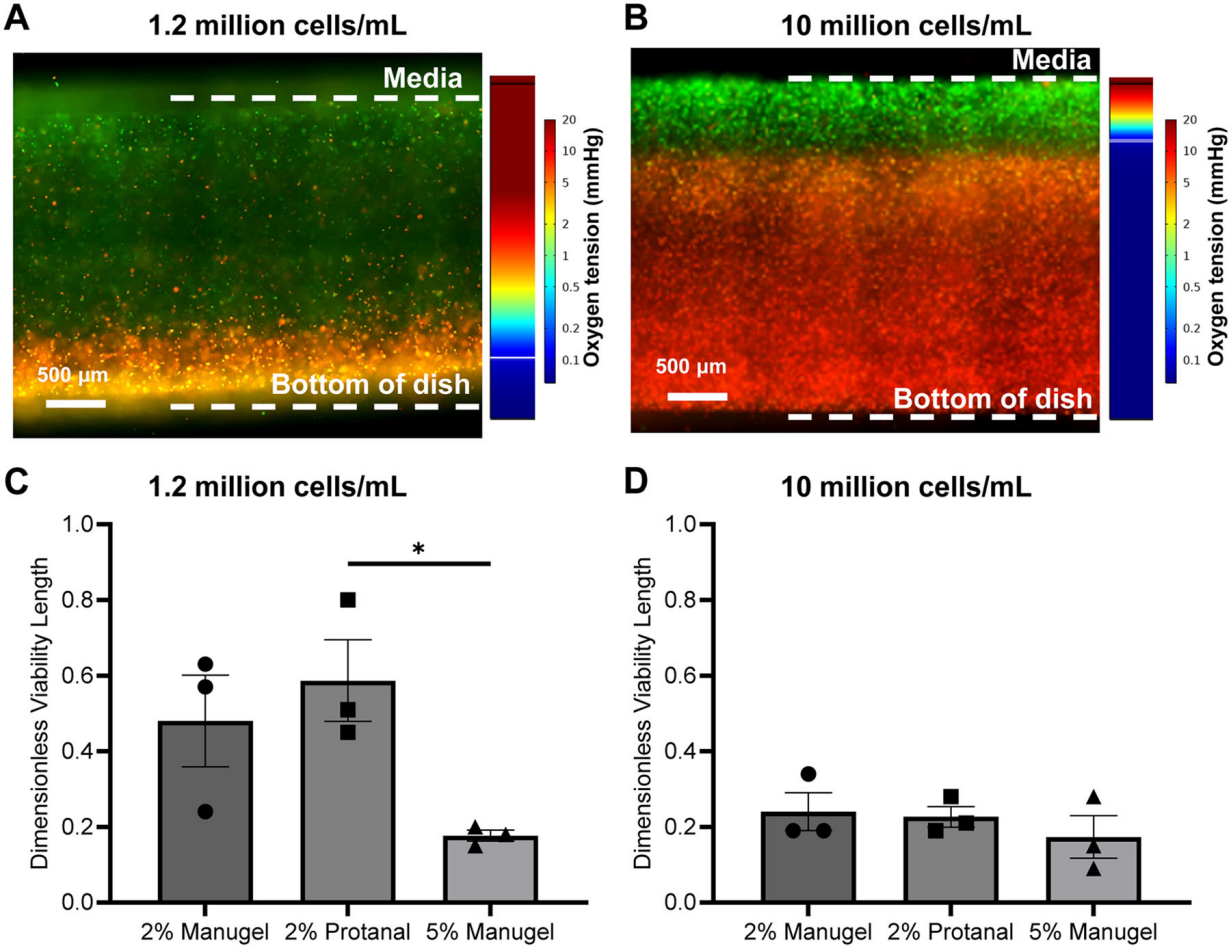
MIN6 viability at different seeding densities in alginate slabs. (A‐B) Representative cross‐section of live (green) and dead (red) MIN6 cells 24 h after seeding at (A) 1.2 million cells/mL or (B) 10 million cells/mL alginate. Colour legends on the right‐hand side represent simulated (COMSOL Multiphysics) pO₂ distribution based on oxygen diffusion and cellular consumption parameters. The white line represents the isocontour where pO₂ = 0.1 mmHg, the threshold below which oxygen tension that delimited the cutoff between viable and dead fronts on average in our data set. (C‐D) Dimensionless viability of MIN6 cells at a concentration of (C) 1.2 million cells/mL or (D) 10 million cells/mL alginate. Corresponds to the length of the green portion of the slab (live cells) over the total portion. *p ≤ 0.05. Error bars: SEM, *N* = 3 experimental replicates.

To determine the relationship between diffusion and cell behaviour, the viability of MIN6 cells in a 1D diffusion slab geometry was evaluated. The experimental viability of MIN6 cells at two different cell fractions is presented in Figure 8.

The dimensionless viable length is the ratio of depth of living cells over the entire depth of the slab. The depth of living cells is determined by measuring the length from the top of the slab to where the living (green) cells abruptly changes to dead (red) cells as shown in Figure 8A and Figure 8B. The abrupt change in cell viability is associated with the existence of an oxygen concentration threshold required for cell survival, despite the oxygen profile being continuous. This potentially abrupt transition is likely associated with a critical oxygen concentration threshold (*C*_*crit*_). This threshold value can be identified from the literature (Cao et al. 2020; Johnson et al. 2009). The difference between hydrogels at high cell fractions is less noticeable (Figure 8D) compared to at a lower cell fraction (Figure 8C) where a significant difference was noticed among groups (*p* < 0.05). As predicted by the trends in the effectiveness factor, changes in viability would become less noticeable at a higher Thiele modulus and therefore cell fraction by extension.

Table 2 presents the effectiveness factors and Thiele moduli for all hydrogels, calculated at the cell fractions used in the experiments shown in Figure 8. As expected, at a concentration of 10 million cells/mL, the differences in effectiveness factor between groups are minimal, due to the high Thiele modulus being much larger than the critical value. However, at a concentration of 1.2 million cells/mL, significant differences are observed: 2% Protanal reportedly achieves an effectiveness factor of 1, while 5% Manugel theoretically achieves only an effectiveness factor of 0.7 at the same cell fraction.

**Table 2:** Thiele modulus and effectiveness factor at 1.2 million cells/mL and 10 million cells/mL for MIN6.

Hydrogel	1.2 million MIN6 cells/mL		10 million MIN6 cells/mL	
	Thiele Modulus (φ)	Effectiveness factor (η)	Thiele Modulus (φ)	Effectiveness factor (η)
2% Manugel	2.31	0.61	6.67	0.21
2% Protanal	1.78	0.79	5.14	0.27
5% Manugel	2.69	0.52	7.79	0.18

## 4 Discussion

In this study, we developed a simple platform to assess oxygen diffusion coefficient in hydrogels such as alginate. Oxygen diffusion coefficients determined using this setup were applied to predict the effect of beta cell seeding density on cell survival, which correlated with simplified unidimensional oxygen diffusion models.

First, the system was validated using water at 37°C as a reference, resulting in an experimental diffusion coefficient of 3.2 × 10^−5^ cm^2^s^−1^ ± 0.5 × 10^−5^ cm^2^s^−1^, which falls within the range of documented values ranging between 2.6 × 10^−5^ cm^2^s^−1^ [42] and 3.83 × 10^−5^ cm^2^s^−1^ (Androjna et al. 2008). Experimental estimates were obtained through transient counter‐diffusion of oxygen after a step change from air to pure nitrogen applied to the system. Experimental trends deviated from predicted curves for unidirectional diffusion‐driven mass transport models, which is characteristic of larger Peclet number systems (Ganaie et al. 2013). Accounting for advection could improve the accuracy of measured diffusion coefficients for fluids such as water. However, advection should be less problematic with hydrogels where fluid motion is hindered by the gel material. The geometry, gas flow, or temperature control strategies could potentially be refined to further reduce sources of error and advection effects.

The system was then applied to various formulations of alginate to determine their diffusion properties. Interestingly, the different formulations of alginate exhibited a much more significant impact on the measured diffusivity coefficient than the concentration of alginate itself. This observation suggests that polymer and gel properties such as the molecular weight distribution, block structure, and degree of crosslinking, can profoundly influence the diffusion characteristics. Since the gels were prepared through internal gelation, the formation of CO_2_ bubbles could also impact the gel rheological and gas diffusion properties. Despite the chemical composition and chain lengths of the different alginate types being very similar, subtle variations in the microstructure and crosslinking density not captured by bulk composition analysis may have led to changes in polymer microstructure that impact oxygen diffusion properties. This finding emphasizes the need for experimental measurement of the diffusion coefficient, as theoretical predictions based solely on concentration or viscosity may overlook critical microstructural differences that affect transport properties. Such variations in the microstructure can lead to discrepancies between estimated and actual diffusion coefficients, which can be critical in applications where precise control over diffusion is required.

The effectiveness factor (η) is a nondimensional number between 0 and 1 that helps understand the impact on mass transport limitations on a chemical reaction – in this case oxygen consumption associated with the viable fraction of cells. Based on low reported Monod constants as compared to oxygen concentrations typically encountered for beta cell encapsulation systems, a simple 0‐order reaction kinetics model was applied, whereby cell death would occur below a critical oxygen concentration. The effect of diffusion on cell viability was quantified by live/dead staining and related to the Thiele modulus φ – a dimensionless number commonly used in (bio)chemical engineering. A cell encapsulation system greatly limited by oxygen diffusion would be associated with a high Thiele modulus and a low effectiveness factor. At high cell fractions (10 million MIN6 cells/mL) where the Thiele modulus is large (φ»φ_crit_), cells become hypoxic because oxygen cannot diffuse through the material fast enough to meet consumption demands, leading to cell death and significantly reduced effectiveness (η«1). In the live/dead experiment (Figure 8), the oxygen within the material is not replenishing fast enough and decreases rapidly as a function of depth resulting in a very sharp live/dead cut‐off (η«1). As seen in Figure 8D, the difference between the effectiveness factors for different materials becomes smaller when Thiele moduli are large. At a lower cell fraction (1.2 million cells/mL), differences between the materials become more obvious showing a significant difference between 2% Protanal and 5% Manugel (Figure 8C). In other words, small changes in oxygen diffusion coefficients of the immobilization material will have greater impact when cell concentrations are lower and the system approaches full viability (η near transition to η = 1).

Dimensionless analyses through Thiele modulus and effectiveness factors are useful in designing islet encapsulation and other immobilized cell culture or transplantation devices. Like MIN6 cells, islets are regarded as a cell type with high oxygen demand – natively they account for 10% of the blood flow in pancreas while only making up 1%–2% of the cell mass (Peiris et al. 2014). For macroencapsulation devices where high cell concentrations are applied (~600,000 IEQ for a 60 kg person) (Lablanche et al. 2021) in small devices (< 50 mL), Thiele moduli will likely be high. In these conditions, small differences in immobilization material oxygen diffusion coefficient will have less impact but significant graft losses should be expected (low effectiveness factor). To achieve effectiveness factors and graft viability near unity, the Thiele modulus and oxygen diffusion coefficient of the immobilization material should be considered. For instance, in the case of 2% Protanal with an effectiveness factor (η) of 0.9, a maximum hydrogel thickness of 0.86 mm would be needed to support a cell density of 50 × 10⁶ cells/mL of human islets. In these cases, the choice of encapsulation material and its corresponding oxygen diffusion coefficient becomes increasingly critical, as the system may behave similarly to the scenario observed in Figure 8C with MIN6 cells at lower cell densities. A more diffusive material could enable a smaller device with equivalent performance and the same therapeutic insulin dose. Alternatively, diffusion distances could be minimized through advection (S. A. et al. 2022; Liang et al. 2021). Simple mass transfer models should be applied early in encapsulation device design and material selection to set constraints and circumvent failure upon scaling from rodents to clinical applications.

## 5 Conclusion

A simple method to measure the oxygen diffusion coefficient was developed for liquids and hydrogels commonly used for cellular encapsulation. The system was validated and measured the diffusion coefficient of water as 3.2 × 10^−5^ ± 0.5 × 10^−5^ cm^2^s^−1^ at 37°C which is within the range of what is currently documented in literature. The system measured the diffusion coefficient of three different formulations of hydrogels (2% Manugel, 2% Protanal, and 5% Manugel). Our study demonstrates a clear correlation between the loss modulus of hydrogels and their effective diffusion coefficients, with lower loss modulus values corresponding to higher diffusion rates. These findings emphasize the critical role of rheological properties in influencing diffusion behavior in hydrogel systems. Moreover, measuring the oxygen diffusivity experimentally allows a more predictive approximation of Thiele and effectiveness factors which are useful dimensionless numbers for encapsulation device sizing and predicting trends is cellular viability.

## Supporting information

20250519 Ebrahimi Orimi and Champion Supplementary.

20221017 Boundary condition check 2D fine.

20220912 2% manugel MIN6 slab model LD KC LG.

20220529 Cap glass cross flow KC.

## Data Availability

The data that support the findings of this study are available from the corresponding author upon reasonable request.
