# Supplementary material for: An Accessible Platform to Quantify Oxygen Diffusion in Cell‐Laden Hydrogels and Its Application to Alginate‐Immobilized Pancreatic Beta Cells
Source: Biotechnol Bioeng. 2025 Nov 11;123(2):348–62. doi: 10.1002/bit.70095 (PMC12779220; doi:10.1002/bit.70095)
Supplement: Supplementary file 1 — 20250519 Ebrahimi Orimi and Champion Supplementary. [file BIT-123-348-s004.pdf]

An accessible platform to quantify oxygen diffusion in cell-laden hydrogels and its application to alginate-immobilized pancreatic beta cells

Hamid Ebrahimi Orimi[1]*, Kurtis Champion[1]*, Laurier Gauvin[1], Jonathan Brassard[1], Berit L. Strand[2], Richard Leask[1], Corinne Hoesli[1]

[1]Department of Chemical Engineering, McGill University, Montreal, Quebec, Canada

[2]Department of Biotechnology and Food Science, Norwegian Biopolymer Laboratory (NOBIPOL), Norwegian University of Science and Technology, 7491 Trondheim, Norway

*Equal contribution

Corresponding author:

Corinne Hoesli

Department of Chemical Engineering

McGill University

3610 Rue University, Montreal, Quebec, Canada, H3A 0C5

Email: corinne.hoesli@mcgill.ca

Phone: (+1) 514-398-4275 (4300)

Date: August 19, 2024

1 Technical Diagram of gas flow cap used in diffusion sensing setup

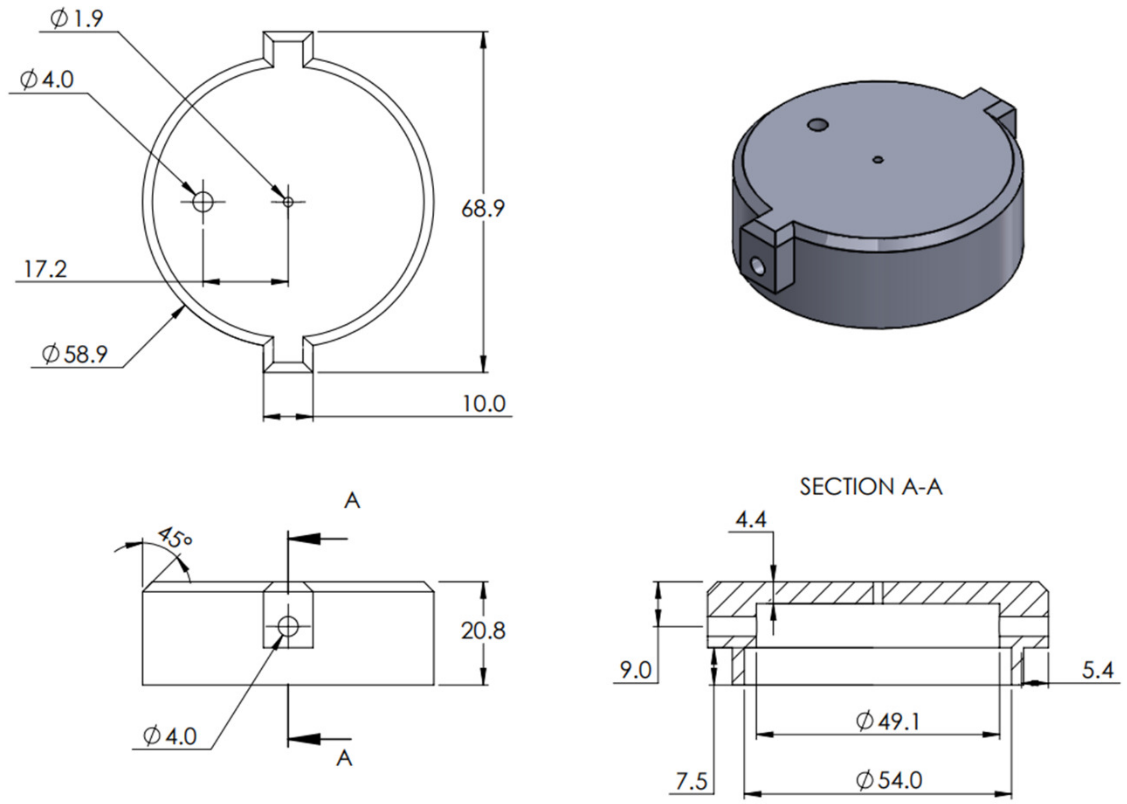

Figure S1. Technical drawing of gas flow cap used in diffusion sensing setup

Units: millimeters (mm)

Note: The STL file will be provided with publication and/or upon request.

Barbed tube hose fittings are attached to both ends; they can be manually forced through when hose fitting has an M4 thread. A Luer lock to hose thread can be attached to the top in the same

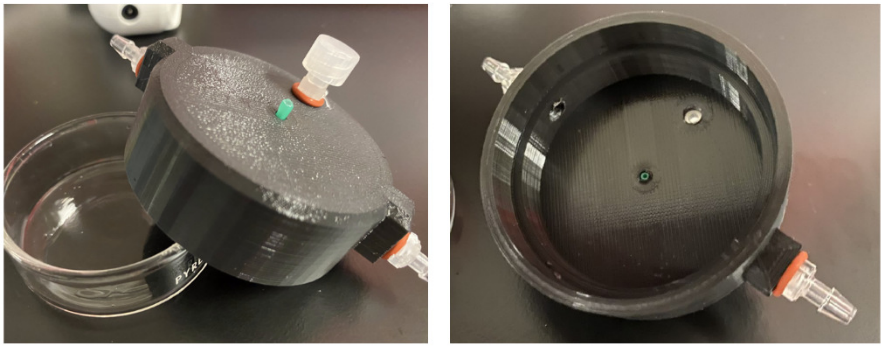

Figure S2. gas flow cap used in diffusion sensing setup

manner. Between all fittings, small 4 mm O-rings were used to create a better seal. The Luer lock connection is sealed with a plastic male Luer lock fixture. Assembly pictures are shown below:

2 Relationship between surface concentration and cell fraction used for calculating the Thiele modulus

The oxygen tension at the surface of the alginate which is below a small layer of media could vary from the oxygen tension at the surface of the media.

A 1-D model following the geometry in Fig 3-1D was developed. In the media portion (total length: 1.5 mm), the diffusivity was assumed to be that of water at 37°C (3.1×10^{-5} cm^2s^{-1}). In the hydrogel, the effective diffusivity (D_{eff}) was calculated using a weighted average of the cell fraction (the X variable), the oxygen diffusivity of tissue (D_{tissue}), and the experimental oxygen diffusivity of the hydrogel in question (2% Manugel, 2% Protanal, or 5% Manugel) referred to as D_{alg}. The oxygen diffusivity of tissue was taken as 1.24×10^{-5} cm^2s^{-1}.

$$D_{eff} = (1 - X)D_{alg} + (X)D_{tissue}$$

Choosing either the OCR for islets or MIN6, a relationship between the cell fraction and surface oxygen tension can be developed for a known geometry.

Note that 0.44 mmHg corresponds to about 6×10^{-4} $mol\cdot m^{-3}$, the Michaelis-Menten coefficient of primary human islets.

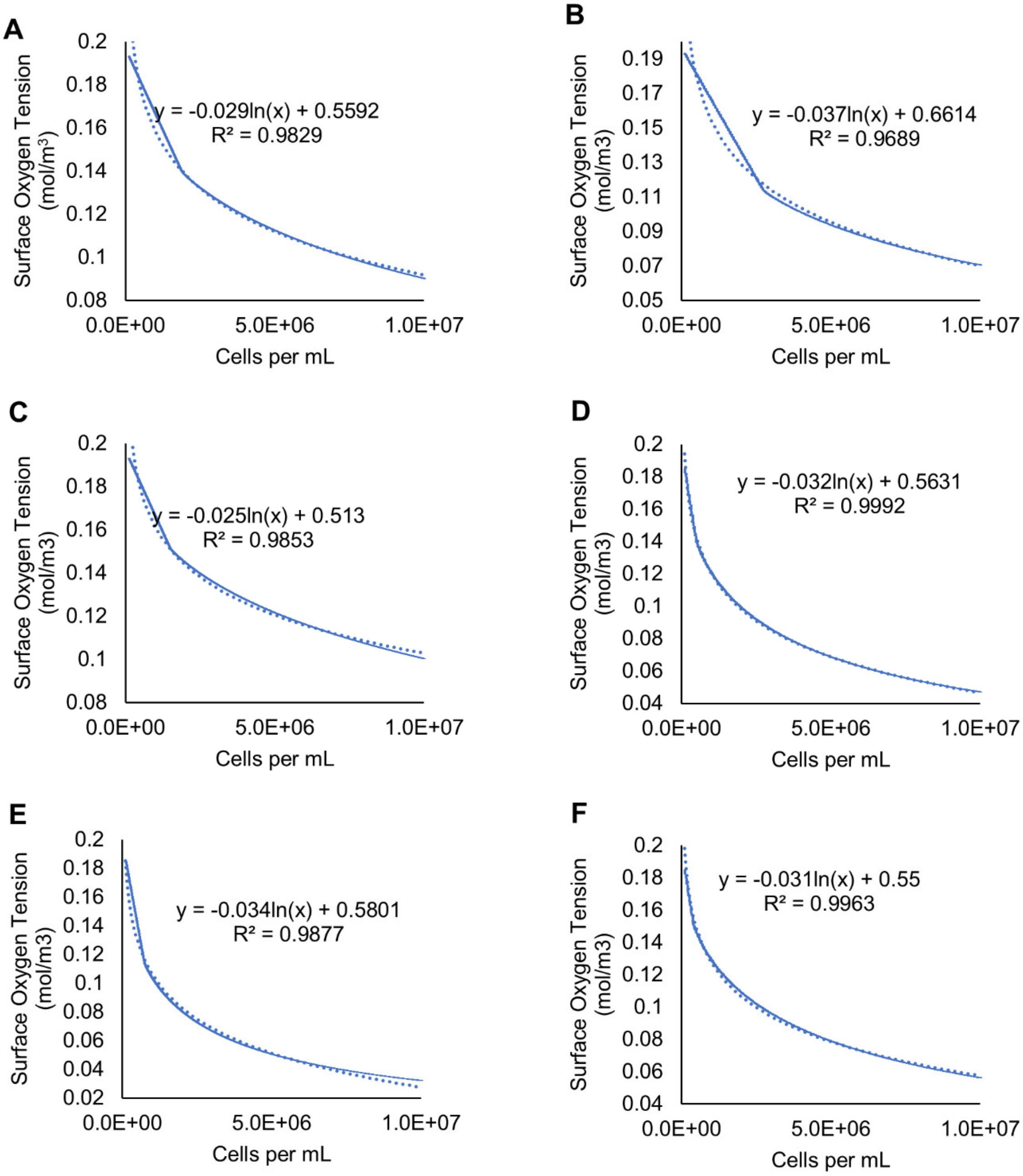

Figure S3. Surface oxygen tension of (A) 2% Manugel (B) 2% Protanal (C) 5% Manugel using human islets (OCR = 0.034 molm^{-3}s^{-1}), and (D) 2% Manugel (E) 2% Protanal (F) 5% Manugel using human islets (OCR = 0.129 molm^{-3}s^{-1})

3 Monitoring oxygen release during oxygen purging process

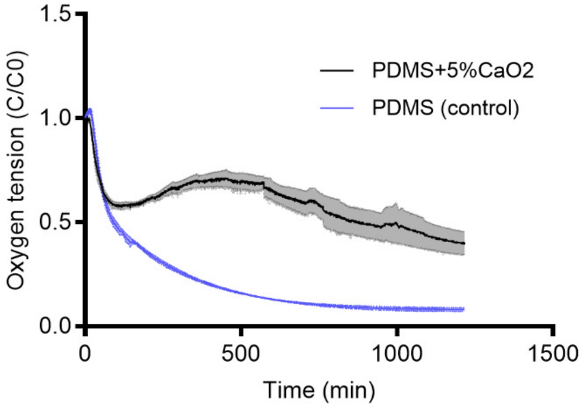

Figure S4. Experimental data of non-dimensional oxygen concentration in air-saturated water on PDMS + 5% CaO_2 (gray) and polydimethylsiloxane (PDMS, blue).

4 Amplitude/Frequency sweep experiment in rheology of hydrogels at room temperature

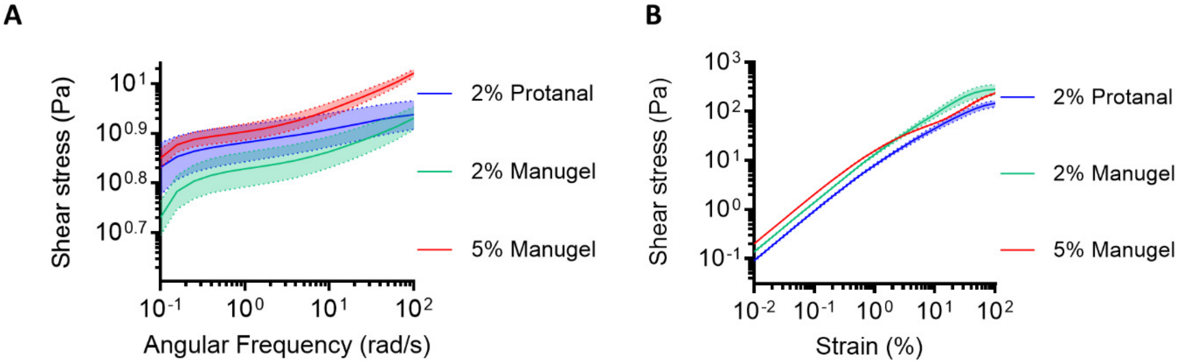

Figure S5. Rheological characterization of hydrogels. (A) Experimental shear stress angular frequency (0.1 to 100 rad/s) (B) Experimental shear stress versus strain (0.01 to 100 %) for 5% w/v Manugel alginate (red zone), 2% w/v Manugel alginate (green zone) and 2% w/v Protanal alginate (blue zone). (Oscillation frequency =10 1/s)

5 Effectiveness factor for 0th order reaction

Using the equation for the Thiele modulus and effectiveness factor, a theoretical estimation of the maximum cell fraction was determined for different hydrogels and cell types (MIN6 cells vs. islets). An effectiveness factor of 0.9 was chosen as the maximum allowable cell fraction since it theoretically corresponds to a fairly good level of oxygenation where the system is not limited by oxygen diffusion.

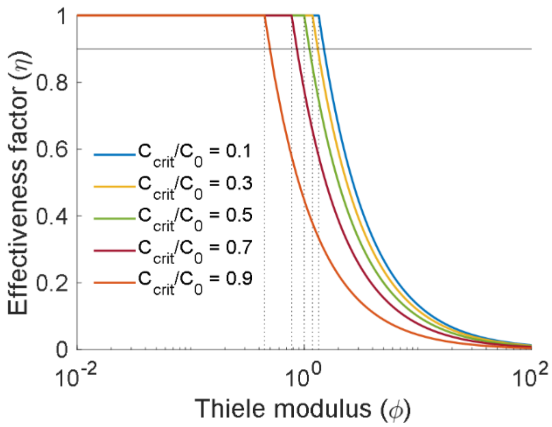

Figure S6. the effectiveness factor vs. Thiele modulus plot for a 0th order reaction. Note that the dashed lines correspond to the critical Thiele modulus (φcrit) where the effectiveness begins decreasing from 1.
